# ERRATUM

**DOI:** 10.21470/1678-9741-2020-0054e

**Published:** 2021

In the article "Permanent Pacemaker Post Cardiac Surgery: where do we Stand?", with DOI code http://dx.doi.org/10.21470/1678-9741-2020-0054, published in the Brazilian Journal of Cardiovascular Surgery, ePub ahead of print on September, 2020, the surname of the second author is misspelled:

In the original version the information was:

Amer Harky^1,2,3^, MBChB, MSc, MRCS; Francesca Gaetta^1^, MD; Arish Noshirwani^1^, MD; Shubhi Gutpa^3^; Muhammed Kermali^4^, MD; Andrew D Muir^1^, MD

The correct information is:

Amer Harky^1,2,3^, MBChB, MSc, MRCS; Francesca Gatta^1^, MD; Arish Noshirwani^1^, MD; Shubhi Gutpa^3^; Muhammed Kermali^4^, MD; Andrew D Muir^1^, MD

